# Food Insecurity Is Associated with Dietary Consumption during the COVID-19 Pandemic: Data from the Korea National Health and Nutrition Examination Survey 2019–2020

**DOI:** 10.3390/nu15030772

**Published:** 2023-02-02

**Authors:** Jeong-Hwa Choi

**Affiliations:** Department of Food Science and Nutrition, Keimyung University, 1095 Dalgubeol-daero, Daegu 42601, Republic of Korea; jhchoi@kmu.ac.kr; Tel.: +82-53-580-5913

**Keywords:** COVID-19, dietary intake, food insecurity, Korean

## Abstract

Coronavirus disease 2019 (COVID-19) has become a pandemic and has affected people’s dietary behaviors, including food insecurity. This study aimed to ascertain whether COVID-19 may alter the prevalence of food insecurity, and if such food security status may be associated with dietary intake among Koreans. The general characteristics, dietary intake and food security status data in the Korea National Health and Nutrition Examination Survey VIII (2019~2020) were analyzed. The prevalence of food insecurity and food group and nutrient consumption by food security status were analyzed before (2019) and after the start of the pandemic (2020). Findings suggested 4.3% of Koreans experienced food insecurity during the first year of the pandemic, yet it did not differ from that in the year before the pandemic. Before COVID-19, there was no significant difference in food group or nutrition consumption by food security status. However, in 2020, the fruit and vitamin C intake of the food-insecure group was significantly lower than that of the food-secure group. Additionally, the food-insecure group’s ratio of subjects who did not meet the recommended level of fruits and the vitamin was higher compared to that of the secure group. In conclusion, COVID-19 did not affect food insecurity status, but did have a negative influence on dietary intake for food-insecure Koreans.

## 1. Introduction

Coronavirus disease 2019 (COVID-19) was declared a pandemic in March 2020 by the World Health Organization (WHO). COVID-19 is now a historic health concern worldwide and has caused approximately 650,879,143 confirmed cases and 6,651,415 related deaths (23rd December 2022) [1]. To prevent the transmission of this infectious respiratory illness, governments have imposed multiple strategies, including social distancing, diagnostic testing, quarantine, vaccination and mobility restriction lockdowns [2,3,4]. These interventions and people’s fear of COVID-19 have massively impacted people’s lives. The global gross domestic product (GDP) in 2020 was −3.3%, which was 5.9% lower than that in 2019 [5]. The total unemployment rate of Organization for Economic Co-operation and Development (OECD) countries was 7.2% in 2020, which was 1.8% higher than that in 2019, before the pandemic [6]. COVID-19 has also influenced not only economic aspects but also people’s daily lifestyles, including dietary behaviors. After the outbreak and during the pandemic, an increase in unhealthy eating was evident among families with lower socioeconomic status [7]. Lower diet quality and nutrient consumption were also reported, especially during the first lockdown period (early 2020) [8,9,10]. Recent meta-analyses suggested that children and adolescents had more high-calorie and high-glycemic foods such as sugary beverages and junk food, and that this was associated with weight gain and obesity [11], although contrasting findings exist [12,13].

Food insecurity is defined as the situation in which individuals experience issues regarding the availability, accessibility, utilization and stability of obtaining nutritious food for a healthy life [14]. Earlier findings suggested that food insecurity is highly associated with lower dietary quality and malnutrition [15]. Individuals with food insecurity had a lower intake of fresh fruits and vegetables but a higher consumption of junk food than those with a secure food supply [16,17,18]. This could lead to food-insecure people, with the interaction of socioeconomic factors, having a higher risk of negative health outcomes, including obesity, hypertension, diabetes and mental health problems [19,20,21,22]. The recent COVID-19 outbreak has also impacted food security status globally. An increased prevalence of household food insecurity was observed during the first wave of the pandemic in the US [23,24], the UK [25], South Asia [26], sub-Saharan Africa and Latin America [27,28]. This is possibly associated with multiple issues, including economic vulnerability, mobility restrictions, and the breakdown of the food supply chain due to the pandemic situation [23,29,30]. However, as described above, this pandemic situation led to contrasting results in food security. The financial and food aid programs during the pandemic may have contributed to ameliorating the inadequate accessibility of food and caused healthier food intake, depending on the time and availability of those measures [31]. This may suggest that the pandemic differentially affects societies, and further health and diet studies that consider different environmental determinants are needed.

The Republic of Korea is in a unique position regarding the control of COVID-19. In February and March 2020, in the early stage of the pandemic, the massive regional outbreaks drew international attention [32], yet the numbers of COVID-19 cases and deaths were smaller in the latter half of 2020 than those in other industrialized countries, such as the US and European countries [2]. Although the economic growth rate of Korea was negative, the country ranked in sixth place among OECD members in 2020 [33]. There were also social gathering restrictions regarding numbers of people, but strict nationwide lockdowns were not imposed in Korea during that time [34,35]. However, similar to other countries, COVID-19 impacted Koreans’ daily lives, including their health-related behaviors. This may play a critical role in further health and disease etiology. Therefore, it is necessary to better understand people’s dietary behaviors during and after the pandemic.

This study aimed to understand Korean individuals’ food security status and associated dietary intake after and during COVID-19, using data from the Korea National Health and Nutrition Examination Survey VIII (KNHANES). The data of KNHANES VIII were collected over 2019 and 2020, i.e., they cover the years before and during the first year of the pandemic. The hypotheses of study are as follows: (1) COVID-19 may affect food insecurity, and (2) food insecurity may be associated with differential dietary intake during the first year of the pandemic. To determine these, analyses were performed to ascertain the prevalence of food insecurity in 2019 and 2020, and to examine any differences that may be evident in food and nutrition intake, taking into account the pandemic and food security status.

## 2. Subjects and Methods

### 2.1. Cohort and Data Description

The present study was performed using data from KNHANES VIII (2019 and 2020). The KNHANES is one of the largest health surveys and is controlled and conducted by the Korea Centers for Disease Control and Prevention (KCDC) and the Ministry of Health and Welfare [36]. The KNHANES was based on a cross-sectional and population-based study that applied a complex, stratified, multistage probability cluster sampling method. A total of 12955 individuals with dietary data were in KNHANES VIII (2019 and 2020). Among these participants, subjects were excluded based on the following criteria. Subjects with implausible daily energy intake (<500 or >5000, *n* = 207), aged under 19 (*n* = 2354), and people without information on lifestyle factors (household income, education, marital/cohabitation status, smoking and drinking status, *n* = 1214), anthropometric data (*n* = 87), food insecurity level (*n* = 23). Additionally, subjects with dietary therapy (*n* = 2681) and those who were lactating or pregnant (*n* = 46) were also excluded. Finally, 3528 out of 6343 subjects in 2019 and 2815 in 2020 were analyzed for this study. The KNHANES VIII was approved by the Institutional Review Board (IRB) of the KCDC (2018-01-03-C-A, 2018-01-03-2C-A), and this study was also reviewed by the IRB of Keimyung University, Korea (40525-202204-HR-014-01).

### 2.2. General and Health-Related Behavior Information Data

General descriptive data (age, sex, household income, educational level, marital/cohabitation status) were collected by face-to-face interviews by trained staff, and health-related behavior (alcohol and tobacco use, practice of aerobic exercise) was assessed by a self-administered questionnaire. The household income status of the study subjects was classified into quartiles: low-middle, middle, middle-high and high. The years of schooling were also grouped into four groups: elementary school and below, middle school, high school and college and above. Alcohol and tobacco use were defined as two levels: currently using (yes) and not using (no). Marital/cohabitation status was grouped into two categories: married/living together with a partner and single (separated, bereavement, divorced and unmarried). The practice of aerobic exercise was defined as two levels. If the subject practiced moderate-intensity activity for more than 150 min/week or vigorous-intensity activity for more than 75 min/week, or either moderate- or vigorous-intensity activity for those defined lengths of time (2 min of moderate-intensity activity was considered 1 min of vigorous-intensity activity), then the answer was “Yes”; otherwise, the answer was “No”.

### 2.3. Anthropometric Data

Body measurements, including height and weight, were conducted by professionals. Body mass index (BMI) was computed as weight in kilograms by squared height in meters (kg/m^2^).

### 2.4. Dietary Data

A nutrition survey was conducted by a dietitian in a face-to-face interview. The 24 h recall method was adapted to collect dietary intake information, and this was converted into food and nutritional intake using the Korean Foods and Nutrients Database of the Rural Development Administration [37]. The types of food consumed by participants were collected and classified into twenty groups. These were further grouped into twelve for this study, considering Koreans’ dietary culture: grains, potatoes, sugars, fruits, vegetables and mushrooms, seaweeds, meats, seafood, eggs, dairy, legumes and nuts, and oils. The following types of food were excluded due to rarity and accuracy concerns: condiments, beverages, alcoholic drinks, and other plants and animals (certain types of oriental medicines or health supplements).

The reference level of the recommended consumption of fruits and selected nutrients was also determined following the Dietary Reference Intakes for Koreans (KDRI), taking into account the subject’s age and sex [38]. A total of 9 nutrients (3 macro- and 5 nutrients that were potentially associated with fruit intake) were evaluated, taking into account food security status. Additionally, subjects’ various dietary intakes for those fruits and nutrients were evaluated with references from the KDRI. For the estimation of fruit consumption, the following criteria were applied. Males aged 19–64 and 65 and older are recommended to have 3 and 2 servings of fruits per day, respectively. In those age groups, females are suggested to have 2 and 1 servings, respectively. Each fruit serving size was estimated to be 100 g, so if an individual’s fruit intake was equal to or over those recommended levels, the subject was considered to have adequate fruit intake. For the estimation of the subject’s nutrient intake, the following references were applied: for protein, vitamin C and folate, the estimated average requirement was used; for dietary fiber and potassium, the adequate intake was used.

### 2.5. Household Food Security Data

The household food security status was estimated using eighteen multiple-choice questions in the KNHANES developed from the U.S. Household Food Security/Hunger Survey Module with modifications [39]. The questionnaire consisted of three parts: overall household, adult- and child-based. If the participants gave an affirmative answer, then the question had a score of one; otherwise, it had a score of zero. The total score was calculated as the sum of the scores, and the food security status was classified into two levels, secure or insecure, as follows: if a subject was from a household with/without children and the total score was equal to or below 2, then they were defined as food-secure and food-insecure otherwise.

### 2.6. Statistical Analyses

All statistical tests were performed with SAS 9.4 (Version 6.2, SAS Institute, Inc., Cary, NC, USA). For the analyses, complex sampling methods and weights were considered following the KNHANES design. The descriptive data are presented as numbers and percentages for categorical variables, and the means and standard errors for continuous variables by food security status. The PROC SURVEYFREQ and PROC SURVEYMEANS commands were used to describe the differences in those variables. The comparisons to examine the differences in food and nutrition intake by food security level were conducted using the PROC SURVEYREG command with the consideration of the subject’s various characteristics. The distribution of individuals who satisfied the recommended fruit and nutrition intake level by food security status was determined using PROC SURVEYFREQ. A *p* < 0.0018 was considered statistically significant considering the multiple comparison issue (0.05/28: number of dietary variables tested).

## 3. Results

### 3.1. The Prevalence of Food Insecurity in 2019 and 2020

The general information of the study cohort is described in Table 1 by survey year and food security status. In 2019, before COVID-19, approximately 3.8% of Koreans were food-insecure. There was a 0.5% increase for subjects with food insecurity in 2020, yet the prevalence of food insecurity did not differ by year (*p* = 0.4664). Several general characteristics, including household income and years of schooling, were associated with the status of food security in both years. Subjects with lower income and education levels were more likely to be in the food-insecure group (all *p* ≤ 0.0001). However, several differences were also evident by the time of the data collection. Before the pandemic, food insecurity status was more prevalent among females (*p* = 0.0018) and elderly people (*p* = 0.0003), while in 2020, no strong association with such characteristics was observed. However, marital/cohabitation status was associated with food insecurity in 2020 (*p* = 0.0005). Substance use including alcohol and tobacco and regular exercise were not significantly associated with food insecurity across the data collection period of 2019–2020.

### 3.2. Food Insecurity and Food and Nutrient Intake

Table 2 presents the level of food intake for subjects grouped by food security status in 2019 and 2020. Statistical analyses suggested that no significant difference was evident in food intake by food security status in 2019. However, in 2020, individuals with limited supply and access to food had decisively smaller amounts of fruits than those who did not have food insecurity issues (*p* < 0.0001). Furthermore, the mean consumption of fruits was 36.4% compared to that of the insecurity group. This association was evident from statistical models that included the subjects’ age, sex, household income, marital/cohabitation status, physical exercise, smoking status, alcohol intake, BMI and total energy intake.

Table 3 describes the major and selected nutrient intake associated with fruits in the food-secure and -insecure groups. Analyses showed that in 2019, there was no clear association between food security status and mean nutrient intake. However, in 2020, individuals with limited food security had lower levels of vitamin C (*p* = 0.0001) consumption. No clear difference was observed in potassium, folate or dietary fiber intake between people of different food security levels.

### 3.3. Food Insecurity and Dietary Intake Taking Account of Recommended Level

**Figure 1:**
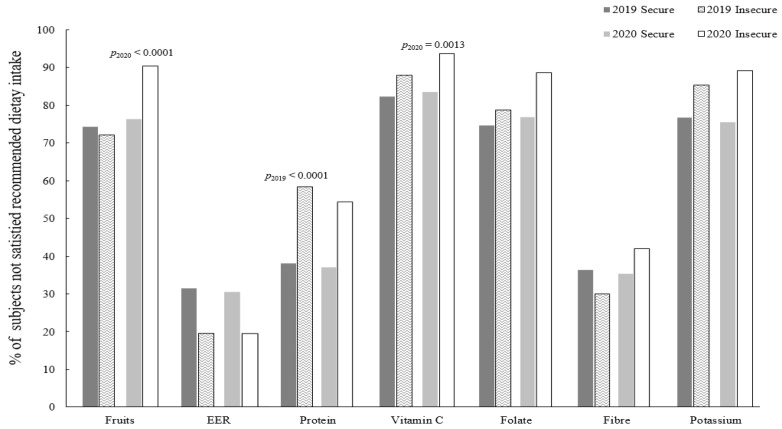
Distribution of the study population that did not meet the recommended Dietary Reference Intakes of selected food and nutrients for Koreans according to food security level. EER; Estimated Energy Requirement. All statistical analyses were performed taking into account the effect of the complex sampling design and weight. *p* values were from Rao-Scott chi-squared tests. *p*_2019_ was from the comparison for dietary intake data in 2019 between food secure and insecure group, and *p*_2020_ was from data in 2020. The recommended level of consumption for each food and nutrient was determined following Dietary Reference Intakes for Koreans taking into account age and sex. Reference levels for protein, vitamin C and folate were the estimated average requirement; for dietary fiber and potassium was the adequate intake.

Figure 1 shows the ratio of subjects who consumed the recommended level of fruit and nutrients, taking into account year and food security level. A total of 8 types of food (fruits) and nutrients (total energy, carbohydrate, protein, vitamin C, folate, fiber and potassium) were analyzed and compared to various recommended levels in the KDRI. Carbohydrates were not included in the figure, because less than 0.01% of subjects consumed lower levels than the recommended levels. The findings suggested that before the pandemic, the proportion of the population that did not consume fewer fruits than the recommended dietary intake did not differ by food security level. However, in 2020, 90.1% of food-insecure individuals did not eat the recommended amount of fruits, which was significantly higher than the percentage of food-secure individuals (75.95%, *p* > 0.0001). A similar trend was also observed in the vitamin C intake pattern in those two groups. In 2019, there was no significant difference in the ratio of subjects who had an inadequate vitamin C intake between the food-secure and food-insecure groups; however, in 2020, that ratio for the food-insecure group was approximately 10% higher than that of the other group (93.73%, *p* = 0.0013) (please see Supplementary Table S1 for detailed information). 

## 4. Discussion

The present study aimed to ascertain whether COVID-19 may be associated with the prevalence of food insecurity among Koreans and whether this may be associated with the consumption of food and nutrients. The study hypotheses were that the pandemic affected food security status, and that Koreans in insecure food supply had a different dietary intake from those in food-secure status. The findings answered such hypotheses: there was no significant increase in the ratio of Koreans with food insecurity. However, after the start of the pandemic, food insecurity status was associated with different dietary consumption levels. 

A number of studies have reported that more people are at risk of food insecurity during the pandemic era [23,40,41]. Although the majority of studies were performed online, before the pandemic, 18.8% of residents of Vermont, US were estimated to be food insecure; however, this increased to 24.8% during the first wave of the pandemic [40]. In other studies in the US, the percentage of food-insecure individuals rose to 22.8%, which was almost double the level expected compared to before the pandemic [41]; in addition, a 17% decrease was also evident in the percentage of families that maintained food security during COVID-19 [42]. However, contrasting findings regarding food insecurity are still evident. In a study of five countries (Australia, Canada, Mexico, the UK and US), it was found that COVID-19 influenced respondents’ dietary behaviors, including their food security, but the impact and the direction of alteration differed between countries. Approximately 68.0 and 41.0% of respondents from Mexico and the US, respectively, reported that their food security status was affected by the pandemic. However, more than 70% of respondents from Australia, Canada, and the UK reported that their food security was not affected by the pandemic; furthermore, 22.6–39.1% of individuals from those five countries thought their diet was actually healthier [31]. COVID-19 has impacted people’s daily lives worldwide; however, the manner and degree of influence vary greatly [31].

In this study, the proportion of Koreans who were food insecure was not significantly changed in 2020 compared to 2019. A few ideas could be suggested to explain this result. First, a number of studies explained that increased food insecurity was mainly due to worsening economic conditions related to COVID-19 [18,23,26,29,43]. As explained above, the Korean economy was in a recession and showed a negative growth rate. However, Korea may have been relatively less affected by COVID-19 than other countries; Korea was ranked 6th among OECD countries in terms of GDP [33]. Second, reduced food security could be associated with physical access to food purchases in addition to economic factors. Repeated long/short-term lockdowns in other countries could have affected food stability as it pertains to the accessibility of food purchases and food acquisition. Although social distancing and gathering measures were imposed in Korea, there has been no substantial lockdown regulation by the government since the outbreak of COVID-19. Lastly, governmental and municipal financial relief and food aid programs may have contributed to Korean food security. However, some minor changes in the population’s characteristics associated with food insecurity were also evident. There were more people with insecure food acquisition and supply status in the higher education and income groups (Table 1). In 2019, approximately 11% of the food-insecure group had a middle-high to high income, but in 2020, more subjects from the same income group had a food security issue (22.6%). In line with this, a similar trend was evident in subjects’ education level: there were more highly educated Koreans in the food-insecure group after the start of the pandemic (in the food-insecure group, the percentage of subjects who had more than a high school education increased by approximately 12%). Furthermore, there were no clear sex or age disparities by food security level compared to before the pandemic. These findings may suggest that COVID-19 seems to have had only a limited effect on Koreans’ diets; no significant change in the prevalence of food insecurity was present in this study. However, there were some substantial changes. Therefore, the findings must be interpreted with caution, and more in-depth and stratified approaches with detailed data are needed.

The findings suggested that some Koreans’ insecure food acquisition status during the first year of COVID-19 was associated with decreased consumption of fruits and a lower percentage of individuals who met the recommended daily intake of vitamin C. Previous studies suggested that food insecurity was often linked to inadequate dietary intake. Food-insecure individuals had less healthy food, which lowered the amount and frequency of fruit and vegetable consumption [16,17]. Differences in nutritional intake were also evidenced by the level of food insecurity [39]. The purchase and intake of fruits are associated with various socioeconomic, lifestyle, cultural and even biological factors [44,45,46]. In Korean cuisine, fruits are consumed between meals or as snacks rather than as a staple food [46]. In line with this, current findings suggested that food insecurity associated with potential economic and/or mobility issues of COVID-19 did not influence the purchase and consumption of staple foods (rice and grains, etc.), but reduced those for fruits. Interestingly, in this study, unlike other reports, Koreans showed a reduced intake of fruits but not of vegetables. This may be because of the type of vegetable dishes Koreans generally consume. Traditional Korean cuisine mainly consists of grains and vegetables, especially pickled (salt, soy sauce, soy paste, or vinegar) forms, such as kimchi and jangachi [47]. Salted vegetables and steamed rice are a very basic Korean meal style. Kimchi is suitable for long-term storage, and it contributes approximately 40% of Koreans’ vegetable intake [48]. This factor may therefore be responsible for the lack of a significant difference in vegetable intake between people of different food security status during the pandemic in the Korean adult population.

The beneficial effects of fruits on human health are well documented. Adequate fruit consumption is protective against multiple chronic degenerative diseases, including hypertension, diabetes mellitus and cancers [49,50]. Therefore, people are recommended to include more fruits in their daily diets. The findings in this study only showed reduced consumption of fruit for a single year, 2020. However, if this altered food intake, including fruits, continues, it may affect diet-related disease development and progression. For instance, in the present study, lower vitamin C intake was more evident in those groups with food security and fruit consumption issues. However, other unmeasured beneficial compounds in food, such as phytochemicals, could affect health and diseases for food-insecure Koreans. For this reason, continued monitoring and supplemental policies for those with food insecurity and inadequate dietary intake are needed.

The present study examined food insecurity status and related dietary intake after the outbreak of the COVID-19 pandemic among Koreans. The findings, which could serve as a reference for public health post-COVID-19, should be interpreted with caution because of the following limitations. First, this study was conducted with KNHANES IIIV, a large-scale, nationwide health and nutritional cohort dataset in Korea. However, this study may not fully represent the features of Koreans, because some participants were excluded during the subject selection procedure (e.g., for lack of lifestyle covariates). These excluded participants and the smaller size of current study population may limit our ability to understand or create associations between COVID-19, food security and dietary intake. Second, in general, the questionnaire is not specifically designed to estimate food security during COVID-19. The statistical models were established considering household income, but for a better understanding, more information, for example, on acute changes in job occupation and income level, is needed. Third, the collection of dietary information was performed using the 24 h recall method. This may not represent the long-term daily dietary intake of Koreans with accuracy. Lastly, this study covered a relatively short period, the first year of the pandemic, 2020. Further analyses with data that fully reflect the COVID-19 era should be conducted.

## 5. Conclusions

In conclusion, the early stage of COVID-19 did not substantially affect food insecurity, but it did influence the dietary intake of Koreans with food insecurity. During the pandemic, individuals with inappropriate food supply and acquisition ate smaller amounts of fruits, and fewer people followed the recommended intake. Food insecurity and the associated reduced intake of fruits and vitamins may affect public health outcomes. Although this finding was from the relatively short period of the COVID-19, the current findings could be referred to as preliminary data. Appropriate health management and policies and constant monitoring for people with food insecurity are required. 

## Figures and Tables

**Table 1:** Descriptive data of the study subjects by food security status.

		2019				2020			
		All(*n* = 3528, 100%)	Food secure (*n* = 3366, 96.2%)	Food insecure (*n* = 162, 3.8%)	*p*	All (*n* = 2815, 100%)	Food secure (*n* = 2686, 95.7%)	Food insecure (*n* = 129, 4.3%)	*p*
Sex	Males	1603 (52.33)	1545 (52.83)	58 (39.60)	0.0018	1252 (51.54)	1202 (51.67)	50 (48.61)	0.5216
	Females	1925 (47.67)	1821 (47.17)	104 (60.40)		1563 (48.46)	1484 (48.33)	79 (51.39)	
Age (years)		48.02 (0.55)	47.76 (0.56)	54.66 (1.83)	0.0003	47.54 (0.59)	47.52 (0.60)	47.97 (2.13)	0.8342
Body mass index (kg/m^2^)		23.57 (0.08)	23.56 (0.09)	23.92 (0.45)	0.4514	23.94 (0.10)	23.94 (0.10)	23.85 (0.68)	0.8930
Household income (tertitle)	Low-middle	696 (14.63)	603 (13.27)	93 (49.62)	<0.0001	523 (14.88)	460 (13.34)	63 (49.18)	<0.0001
	Middle	946 (25.96)	894 (25.45)	52 (39.04)		674 (21.83)	635 (21.55)	39 (28.25)	
	Middle-high	905 (27.78)	892 (28.49)	13 (9.65)		802 (29.52)	781 (30.10)	21 (16.56)	
	High	981 (31.63)	977 (32.80)	4 (1.69)		816 (33.78)	810 (35.01)	6 (6.01)	
Education	Elementary or less	725 (13.75)	659 (13.04)	66 (32.01)	<0.0001	530 (12.63)	489 (12.27)	41 (20.73)	0.0001
	Middle school	331 (7.69)	311 (7.51)	20 (12.26)		312 (8.52)	292 (8.38)	20 (11.59)	
	High school	1149 (35.02)	1095 (34.89)	54 (38.49)		955 (37.77)	906 (37.38)	49 (46.62)	
	College or more	1323 (43.53)	1301 (44.56)	22 (17.24)		1018 (41.07)	999 (41.96)	19 (21.06)	
Alcohol drinking (current)	No	1614 (41.41)	1527 (41.16)	87 (47.89)	0.1928	1353 (43.41)	1274 (42.95)	79 (53.5)	0.0221
	Yes	1914 (58.59)	1839 (58.84)	75 (52.11)		1462 (56.59)	1412 (57.04)	50 (46.41)	
Tobacco smoking (current)	No	2882 (78.25)	2754 (78.40)	128 (74.27)	0.3553	2311 (79.45)	2214 (79.69)	97 (74.02)	0.1804
	Yes	646 (21.75)	612 (21.59)	34 (25.73)		504 (20.55)	472 (20.31)	32 (25.98)	
Marital status	Married/cohabiting	2439 (66.16)	2351 (66.63)	88 (54.19)	0.0108	1838 (62.41)	1781 (63.28)	57 (43.02)	0.0005
	Single	1089 (33.83)	1015 (33.37)	74 (45.80)		977 (37.58)	905 (36.72)	72 (56.97)	
Practice of aerobic exercise	No	2082 (55.95)	1971 (55.53)	111 (66.88)	0.0145	1720 (59.24)	1639 (59.11)	81 (62.10)	0.5948
	Yes	1439 (44.05)	1389 (44.47)	50 (33.12)		1091 (40.76)	1043 (40.89)	48 (37.90)	
Food insecurity score		0.27 (0.03)	0.11 (0.01)	4.57 (0.23)	<0.0001	0.32 (0.03)	0.13 (0.01)	4.55 (0.21)	<0.0001

All statistical analyses were performed taking into account the effect of the complex sampling design and weight. *p* values were from analyses of variance for age, body mass index, and food insecurity score; otherwise, chi-squared tests were used. Age, body mass index, and food insecurity score are presented as the means and SEs or as numbers and weighted percentages.

**Table 2:** Intake level for selected food groups by food security status in 2019 and 2020 (g/day, mean and SE).

		2019		*p*		2020		*p*
	All	Food Secure	Food Insecure		All	Food Secure	Food Insecure	
Grains	281.74 (3.76)	282.5 (3.77)	260.4 (17.15)	0.5497	277.4 (3.74)	277.4 (3.81)	279.0 (17.84)	0.2946
Potatoes	33.36 (1.58)	33.64 (1.62)	26.33 (6.05)	0.4659	31.50 (1.81)	31.56 (1.82)	30.31 (8.69)	0.6895
Sugar	9.50 (0.40)	9.51 (0.41)	9.21 (2.13)	0.3062	8.48 (0.37)	8.50 (0.37)	8.15 (1.87)	0.3506
Fruits	143.9 (4.98)	144.2 (4.98)	135.3 (22.91)	0.5900	130.1 (6.12)	133.9 (6.28)	47.44 (8.90)	<0.0001
Vegetables & Mushroom	305.2 (4.66)	307.8 (4.74)	240.4 (17.80)	0.1215	297.1 (5.21)	299.7 (5.21)	239.9 (19.00)	0.0343
Seaweeds	27.67 (2.31)	27.80 (2.35)	24.22 (9.23)	0.8102	27.23 (2.15)	27.62 (2.24)	18.61 (6.13)	0.4080
Meats	128.0 (3.75)	129.85 (3.89)	82.42 (12.25)	0.5596	128.9 (4.64)	130.0 (4.70)	105.5 (16.60)	0.9276
Seafood	106.2 (3.13)	106.0 (3.13)	110.9 (20.37)	0.1887	97.84 (4.19)	98.63 (4.28)	80.69 (16.44)	0.8973
Eggs	32.97 (1.11)	33.18 (1.15)	27.74 (4.23)	0.7548	31.41 (1.16)	31.69 (1.18)	25.29 (5.02)	0.2450
Dairy	81.02 (2.93)	81.53 (2.97)	67.87 (12.992)	0.9454	77.94 (3.91)	79.19 (4.00)	50.43 (13.27)	0.1079
Legumes & nuts	41.37 (1.70)	41.50 (1.75)	37.98 (6.89)	0.8496	42.31 (2.07)	42.62 (2.14)	35.48 (8.36)	0.8737
Oils	6.75 (0.22)	6.82 (0.22)	4.96 (0.79)	0.7729	6.85 (0.25)	6.96 (0.25)	4.46 (0.61)	0.0250

All statistical analyses were performed taking into account the effect of the complex sampling design and weight. *p* values were from the analysis of covariance between the food-secure and -insecure groups and adjusted for age, sex, household income, marital status, physical activity level, smoking and drinking status, body mass index, and total energy intake.

**Table 3:** Nutrient intake level by food security status in 2019 and 2020 (mean and SE).

		2019		*p*		2020		*p*
	All	Food Secure	Food Insecure		All	Food Secure	Food Insecure	
Energy (Kcal)	1964.0 (20.97)	1976.3 (21.15)	1650.0 (81.42)	0.1844	1931.6 (21.36)	1939.7 (21.33)	1753.4 (106.5)	0.3778
Carbohydrate (g)	285.9 (2.70)	286.9 (2.69)	261.6 (12.44)	0.4699	276.6 (2.76)	276.8 (2.72)	271.4 (17.27)	0.2589
Protein (g)	72.22 (1.00)	72.75 (1.01)	58.67 (4.03)	0.9945	71.29 (1.06)	71.76 (1.05)	60.83 (4.77)	0.2594
Fat (g)	47.88 (0.94)	48.39 (0.95)	35.06 (2.82)	0.8607	49.06 (1.01)	49.44 (1.01)	40.84 (3.96)	0.6168
Dietary fibre (g)	23.75 (0.30)	23.90 (0.30)	20.30 (1.04)	0.4157	24.14 (0.31)	20.30 (1.04)	20.53 (1.46)	0.2120
Folate (µg DFE)	318.3 (3.89)	320.2 (3.89)	271.6 (12.90)	0.3551	309.8 (4.13)	312.0 (4.07)	260.6 (18.46)	0.0168
Vitamin C (mg)	64.79 (1.81)	65.38 (1.87)	49.82 (4.32)	0.3025	65.88 (2.67)	66.95 (2.70)	42.37 (5.66)	0.0001
Potassium (mg)	2759.7 (31.18)	2778.0 (31.31)	2295.9 (122.3)	0.3377	2780.3 (32.69)	2801.1 (32.41)	2323.2 (149.2)	0.0821

DFE; dietary folate equivalents. All statistical analyses were performed taking into account the effect of the complex sampling design and weight. *p* values were from the analysis of covariance between the food-secure and -insecure groups and adjusted for age, sex, household income, marital status, physical activity level, smoking and drinking status, body mass index, and total energy intake, as appropriate.

## Data Availability

The study analyzed the data of KNHANES from the KCDC (https://knhanes.kdca.go.kr/knhanes/eng/index.do, accessed on 1 February 2023).

## Supplementary Materials

The following supporting information can be downloaded at: https://www.mdpi.com/article/10.3390/nu15030772/s1, Table S1. Distribution of study population not satisfied Dietary Reference Intakes of selected food and nutrients for Koreans according to food security level.

## References

[1] World Health Organization WHO Coronavirus (COVID-19) Dashboard. https://www.who.int/emergencies/diseases/novel-coronavirus-2019.

[2] Dighe A., Cattarino L., Cuomo-Dannenburg G., Skarp J., Imai N., Bhatia S., Gaythorpe K.A.M., Ainslie K.E.C., Baguelin M., Bhatt S. (2020). Response to COVID-19 in South Korea and implications for lifting stringent interventions. BMC Med..

[3] Kwon S.L., Oh J. (2022). COVID-19 vaccination program in South Korea: A long journey toward a new normal. Health Policy Technol..

[4] Onyeaka H., Anumudu C.K., Al-Sharify Z.T., Egele-Godswill E., Mbaegbu P. (2021). COVID-19 pandemic: A review of the global lockdown and its far-reaching effects. Sci. Prog..

[5] The World Bank Global Gross Domestic Product. https://data.worldbank.org/indicator/NY.GDP.MKTP.KD.ZG.

[6] Organization for Economic Cooperation and Development Unemployment Rate (Indicator).

[7] McNicholas J., Hammersley M.L., Hopkins S., McDermott S., Plaskett J. (2022). The Impact of COVID-19 Restrictions on the Healthy Eating and Movement Behaviors of 0-12-Year-Old Children in Western Sydney, Australia. Front. Public Health.

[8] Marty L., de Lauzon-Guillain B., Labesse M., Nicklaus S. (2021). Food choice motives and the nutritional quality of diet during the COVID-19 lockdown in France. Appetite.

[9] Del Pozo de la Calle S., Alonso Ledesma I., Nuñez O., Castelló Pastor A., Lope Carvajal V., Fernández de Larrea Baz N., Pérez-Gómez B., Pollán M., Ruiz Moreno E. (2021). Composition and nutritional quality of the diet in Spanish households during the first wave of the COVID-19 Pandemic. Nutrients.

[10] Deschasaux-Tanguy M., Druesne-Pecollo N., Esseddik Y., de Edelenyi F.S., Allès B., Andreeva V.A., Baudry J., Charreire H., Deschamps V., Egnell M. (2021). Diet and physical activity during the coronavirus disease 2019 (COVID-19) lockdown (March-May 2020): Results from the French NutriNet-Santé cohort study. Am. J. Clin. Nutr..

[11] La Fauci G., Montalti M., Di Valerio Z., Gori D., Salomoni M.G., Salussolia A., Soldà G., Guaraldi F. (2022). Obesity and COVID-19 in children and adolescents: Reciprocal detrimental influence-systematic literature review and meta-Analysis. Int. J. Environ. Res. Public Health.

[12] Murphy B., Benson T., McCloat A., Mooney E., Elliott C., Dean M., Lavelle F. (2020). Changes in consumers’ food practices during the COVID-19 lockdown, implications for diet quality and the food system: A Cross-continental comparison. Nutrients.

[13] Benson T., Murphy B., McCloat A., Mooney E., Dean M., Lavelle F. (2022). From the pandemic to the pan: The impact of COVID-19 on parental inclusion of children in cooking activities: A cross-continental survey. Public Health Nutr..

[14] Gibson M. (2012). Food security-a commentary: What is it and why is it so complicated?. Foods.

[15] Pereira M.H.Q., Pereira M., Campos G.C., Molina M.C.B. (2022). Food insecurity and nutritional status among older adults: A systematic review. Nutr. Rev..

[16] Leung C.W., Epel E.S., Ritchie L.D., Crawford P.B., Laraia B.A. (2014). Food insecurity is inversely associated with diet quality of lower-income adults. J. Acad. Nutr. Diet..

[17] Hanson K.L., Connor L.M. (2014). Food insecurity and dietary quality in US adults and children: A systematic review. Am. J. Clin. Nutr..

[18] Jimenez Rincon S., Dou N., Murray-Kolb L.E., Hudy K., Mitchell D.C., Li R., Na M. (2022). Daily food insecurity is associated with diet quality, but not energy intake, in winter and during COVID-19, among low-income adults. Nutr. J..

[19] Militao E.M.A., Salvador E.M., Uthman O.A., Vinberg S., Macassa G. (2022). Food insecurity and health outcomes other than malnutrition in Southern Africa: A descriptive systematic review. Int. J. Environ. Res. Public Health.

[20] Long C.R., Narcisse M.R., Bailey M.M., Rowland B., English E., McElfish P.A. (2022). Food insecurity and chronic diseases among Native Hawaiians and Pacific Islanders in the US: Results of a population-based survey. J. Hunger Environ. Nutr..

[21] O’Neal L.J., Jo A., Scarton L., Bruce M.A. (2022). Food Insecurity Is Associated with mental-physical comorbidities among U.S. adults: NHANES 2013 to 2016. Int. J. Environ. Res. Public Health.

[22] Sun Y., Liu B., Rong S., Du Y., Xu G., Snetselaar L.G., Wallace R.B., Bao W. (2020). Food Insecurity is associated with cardiovascular and all-cause mortality among adults in the United States. J. Am. Heart Assoc..

[23] Litton M.M., Beavers A.W. (2021). The relationship between food security status and fruit and vegetable intake during the COVID-19 pandemic. Nutrients.

[24] Fitzpatrick K.M., Harris C., Drawve G., Willis D.E. (2021). Assessing food insecurity among US adults during the COVID-19 pandemic. J. Hunger Environ. Nutr..

[25] Koltai J., Toffolutti V., McKee M., Stuckler D. (2021). Prevalence and changes in food-related hardships by socioeconomic and demographic groups during the COVID-19 pandemic in the UK: A longitudinal panel study. Lancet Reg. Health Eur..

[26] Shuvo S.D., Hossain M.S., Riazuddin M., Mazumdar S., Roy D. (2022). Factors influencing low-income households’ food insecurity in Bangladesh during the COVID-19 lockdown. PLoS ONE.

[27] Rudin-Rush L., Michler J.D., Josephson A., Bloem J.R. (2022). Food insecurity during the first year of the COVID-19 pandemic in four African countries. Food Policy.

[28] Rogus S., Coakley K.E., Martin S., Gonzales-Pacheco D., Sroka C.J. (2022). Food security, access, and challenges in New Mexico during COVID-19. Curr. Dev. Nutr..

[29] Brown H., Mills S., Albani V. (2022). Socioeconomic risks of food insecurity during the Covid-19 pandemic in the UK: Findings from the Understanding Society Covid Survey. BMC Public Health.

[30] Picchioni F., Goulao L.F., Roberfroid D. (2021). The impact of COVID-19 on diet quality, food security and nutrition in low and middle income countries: A systematic review of the evidence. Clin. Nutr..

[31] Acton R.B., Vanderlee L., Cameron A.J., Goodman S., Jáuregui A., Sacks G., White C.M., White M., Hammond D. (2022). Self-reported impacts of the COVID-19 pandemic on diet-related behaviors and food security in 5 countries: Results from the International Food Policy Study 2020. J. Nutr..

[32] Choi S., Ki M. (2020). Estimating the reproductive number and the outbreak size of COVID-19 in Korea. Epidemiol. Health.

[33] Statistics Korea Main Statistical Indicators in OECD. https://kosis.kr/statHtml/statHtml.do?orgId=101&tblId=DT_2KAAG01&conn_path=I3.

[34] Task Force for Tackling COVID-19 All about Korea’s Response to COVID-19. South Korea. http://www.kdca.go.kr/upload_comm/syview/doc.html?fn=160276224199800.pdf&rs=/upload_comm/docu/0030/.

[35] Ryu S.-K., Chung S.-G. (2021). Korea’s early COVID-19 response: Findings and implications. Int. J. Environ. Res. Public Health.

[36] Oh K., Kim Y., Kweon S., Kim S., Yun S., Park S., Lee Y.K., Kim Y., Park O., Jeong E.K. (2021). Korea National Health and Nutrition Examination Survey, 20th anniversary: Accomplishments and future directions. Epidemiol. Health.

[37] National Institute of Agricultural Sciences (2017). Korean Food Composition Table.

[38] Ministry of Health and Welfare. The Korean Nutrition Society (2020). Dietary Reference Intakes for Koreans 2020.

[39] Kim K., Hong S.A., Kwon S.O., Choi B.Y., Kim G.-Y., Oh S.-Y. (2011). Validation of food security measures for the Korean National Health and Nutrition Examination Survey. Korean J. Community Nutr..

[40] Niles M.T., Bertmann F., Belarmino E.H., Wentworth T., Biehl E., Neff R. (2020). The early food insecurity impacts of COVID-19. Nutrients.

[41] Schanzenbach D.P.A. (2020). How Much Has Food Insecurity Risen?. Evidence from the Census Household Pulse Survey.

[42] Adams E.L., Caccavale L.J., Smith D., Bean M.K. (2020). Food insecurity, the home food environment, and parent feeding practices in the era of COVID-19. Obesity.

[43] Dasgupta S., Robinson E.J.Z. (2022). Impact of COVID-19 on food insecurity using multiple waves of high frequency household surveys. Sci. Rep..

[44] Dehghan M., Akhtar-Danesh N., Merchant A.T. (2011). Factors associated with fruit and vegetable consumption among adults. J. Hum. Nutr. Diet..

[45] Choi J.H. (2019). Variation in the *TAS2R38* Bitterness Receptor Gene Was Associated with Food Consumption and Obesity Risk in Koreans. Nutrients.

[46] Choi S.A., Chung S.S., Rho J.O. (2021). Analysis of fruit consumption and the Korean Healthy Eating Index of adults using the 2018 Korea National Health and Nutrition Examination Survey. J. Korean Soc. Food Sci. Nutr..

[47] Kim S.H., Kim M.S., Lee M.S., Park Y.S., Lee H.J., Kang S.-A., Lee H.S., Lee K.-E., Yang H.J., Kim M.J. (2016). Korean diet: Characteristics and historical background. J. Ethn. Foods.

[48] Kim S.Y., Freeland-Graves J.H., Kim H.J. (2021). Twenty-year trends in vegetable consumption by preparation method and eating location for Korean population from 1998 to 2017. Br. J. Nutr..

[49] Wu Y., Zhang D., Jiang X., Jiang W. (2015). Fruit and vegetable consumption and risk of type 2 diabetes mellitus: A dose-response meta-analysis of prospective cohort studies. Nutr. Metab. Cardiovasc. Dis..

[50] Wang X., Ouyang Y., Liu J., Zhu M., Zhao G., Bao W., Hu F.B. (2014). Fruit and vegetable consumption and mortality from all causes, cardiovascular disease, and cancer: Systematic review and dose-response meta-analysis of prospective cohort studies. BMJ.

