# Supplementary material for: Food Insecurity Is Associated with Dietary Consumption during the COVID-19 Pandemic: Data from the Korea National Health and Nutrition Examination Survey 2019–2020
Source: Nutrients. 2023 Feb 2;15(3):772. doi: 10.3390/nu15030772 (PMC9919441; doi:10.3390/nu15030772)
Supplement: Supplementary file 1 [file nutrients-15-00772-s001.zip › nutrients-2159236-supplementary.pdf]

Supplementary Table S1. Distribution of study population not satisfied Dietary Reference Intakes of selected food and nutrients for Koreans according to food security level.

	2019			2020		
	Secure (*n*, %)	Insecure (*n*, %)	*p*	Secure (*n*, %)	Insecure (*n*, %)	*p*
Fruit	2387 (74.25)	116 (72.11)	0.6607	1938 (75.95)	107 (90.07)	<.0001
EER	1041 (31.55)	34 (19.50)	0.0032	776 (30.07)	21 (19.25)	0.0366
Protein	1397 (38.05)	92 (58.33)	<.0001	1104 (37.77)	77 (55.43)	0.0018
Carbohydrate	1 (0.01)	0	-	0	0	-
Vitamin C	2768 (82.31)	139 (87.98)	0.0781	2228 (83.45)	119 (93.73)	0.0013
Folate	2503 (74.63)	127 (78.78)	0.2609	2025 (76.45)	115 (88.30)	0.0147
Dietary fiber	1054 (36.35)	42 (30.00)	0.1991	791 (35.50)	49 (42.10)	0.2212
Potassium	2592 (76.75)	137 (85.40)	0.0181	2024 (74.58)	117 (88.03)	0.0107

EER; Estimated Energy Requirement.

All statistical analyses performed taking account of the effect of complex sampling design and weight. *P* values were from Rao-Scott Chi-Squared tests. Recommended level of each food and nutrition consumption was determined following Dietary Reference Intakes for Koreans taking account of age and sex. Reference levels for protein, carbohydrate, vitamin C and folate were Estimated Average Requirement; for dietary fibre and potassium was Adequate Intake.
